# Mathematical modeling and analysis for the co-infection of COVID-19 and tuberculosis

**DOI:** 10.1016/j.heliyon.2022.e11195

**Published:** 2022-10-20

**Authors:** Kassahun Getnet Mekonen, Legesse Lemecha Obsu

**Affiliations:** a Department of Mathematics, Hawassa University, Hawassa, Ethiopia; b Department of Applied Mathematics, Adama Science and Technology University, Adama, Ethiopia

**Keywords:** TB, COVID-19, Co-infection, Stability analysis, Sensitivity

## Abstract

We developed a TB-COVID-19 co-infection epidemic model using a non-linear dynamical system by subdividing the human population into seven compartments. The biological well-posedness of the formulated mathematical model was studied via proving properties like boundedness of solutions, no-negativity, and the solution's dependence on the initial data. We then computed the reproduction numbers separately for TB and COVID-19 sub-models. The criterion for stability conditions for stationary points was examined. The basic reproduction number of sub-models used to suggest the mitigation and persistence of the diseases. Qualitative analysis of the sub-models revealed that the disease-free stationary points are both locally and globally stable provided the respective reproduction numbers are smaller than unit. The endemic stationary points for each sub-models were globally stable if their respective basic reproduction numbers are greater than unit. In each sub-model, we performed an analysis of sensitive parameters concerning the corresponding reproduction numbers. Results from sensitivity indices of the parameters revealed that deceasing contact rate and increasing the transferring rates from the latent stage to an infected class of individuals leads to mitigating the two diseases and their co-infections. We have also studied the analytical behavior of the full co-infection model by deriving the equilibrium points and investigating the conditions of their stability. The numerical experiments of the proposed co-infection model agree with the findings in the analytical results.

## 1 Introduction

Coronavirus disease (COVID-19) is a respiring virus that spreads via contact with saliva droplets released during coughing or sneezing of an infected person [1]. Due to COVID-19, more than 5 million deaths and 269 million infections have been reported since 12 December 2021 [2]. While, Tuberculosis (TB) is an old contagious disease acquired by *Mycobacterium* bacteria. It spreads when an active TB patient coughs, sneezes, or speaks and someone else inhales the expelled droplets, which contain TB bacteria. Worldwide, an estimate of 9.9 (ranging, from 8.9–11.1) million individuals were sick and 1.5 million life were lost due to TB in 2020 [3].

TB and COVID-19 are contagious disease which primarily attack the lungs. The experimental data on TB role in spreading COVID-19 remains inadequate, however, it was predicted that people co-infected with both disease might obtained substandard treatment which may lead to critical conditions [4]. The clinical symptoms of both diseases include fever, cough, chest pain, and difficulty in breathing [4], [5]. TB-COVID-19 co-infection might occur whenever peoples are infected simultaneously with these diseases. In this regard, clinical evidence reveals that COVID-19 infection may happen at any status of TB stage [6]. Further, the study reported in [7] confirmed that latent and active TB are the main risk factors to increase the spread of COVID-19 in the community. Besides, the study presented in [8] reveals that people at the stage of active or latent TB cases are more vulnerable to COVID-19. In this report, the authors stated that the symptom continuances of the disease on the patients are more rapid and severe. It was estimated that the mortality is higher than 12.3% in the cases of co-infections compared to COVID-19 alone [9].

Many dynamical models have been developed and analyzed to identify most important parameters that helps to forecast the trends of infectious disease and its control mechanisms [10]. With aim different mathematical models were formulated and analyzed to investigate the pandemic of COVID-19 in [11], [12], [13], [14], [15], and references cited therein. Similarly, many TB dynamical models are also studied to proposed its mitigation strategies [16], [17], [18], [19].

Studies on the co-dynamics of COVID-19 and other infectious disease are also studied in various literature. For instance, control induced dynamical model was formulated and analyzed to examine Cholera-COVID-19 in [20]. Similarly, TB-HIV/AIDS can be found in [21], [22], Dengue-COVID-19 [23], and TB-COVID-19 in [24], [25]. In [24], Marimthu et al. estimated the number of TB-COVID-19 co-infected people with and without intervention mechanisms. The results of their study revealed that the peak of the co-infection had occurred relatively in a small time with no intervention compared to that of interventions. Their model estimated that about 20,880 TB-COVID-19 cases will occur on the epidemic peak day when intervention mechanisms are imposed and 27,968 cases will happen in the absence of proper interventions. In [25], an Atangana-Baleanu type mathematical model was considered to investigate TB-COVID-19 co-infection using fractional-order derivative. Their simulations studies indicated that the TB-COVID-19 co-infection becomes decline via deceasing the TB latent infected people who are at risk with COVID-19.

Motivated by the discussions above, we formulated a mathematical model governing TB-COVID-19 co-infection to study their co-dynamics. For this purpose, the population is grouped into seven disjoint compartments. We included the TB latent compartment to study the greater risk of people progressed to active TB disease, and the exposed COVID-19 compartment to assess the risk of its progression to infected class following initial stages. Besides, the roles of the contact rates of both diseases and the transfer rates are studied. The sensitivity of parameters with reproduction numbers for the sub-models was also examined to identify the most sensitive parameters. In addition, the role of TB in the spread of COVID-19 pandemic was discussed. Furthermore, to show the local stability of equilibrium points, the linearization approach was utilized while Lyapunov technique was applied to investigate the corresponding global stability.

Our study differs from the Omame et al. paper [25], that is, in our study, the dynamics are described in terms of the integer time derivative that has a widely understandable geometric and physical descriptions. It is appropriate for dynamic models, and describes how the state of the system changes as time changes. In contrary, Omame et al. in their study utilized fractional derivative model with the help of Atangana-Baleanu derivative to describe the memory effect and temporal change.

The organization of the paper is described as follows. Assumptions to model COVID-19-TB co-infection were formulated, and well analyzed in Section 2. Qualitative investigation of the developed model is performed by finding stationary points of the sub-models, and the full model in Section 3. Numerical simulation were given in Section 4. Finally, conclusion and recommendations were provided in Section 5.

## 2 Model formulation

A model for the co-dynamics of TB-COVID-19 was formulated by subdividing the population into seven compartmental states. Namely the subdivisions are: susceptible individuals (*S*), TB infected people in a latent stage ($L_{T}$), individuals in the active TB state ($I_{T}$), COVID-19 exposed ($E_{C}$), symptomatically ill people with COVID-19 ($I_{C}$), COVID-19-TB co-infected class ($I_{TC}$), and people recovered from both diseases (*R*). The susceptible class of individuals are members of a population those are at risk of becoming infected by a disease, while recovered individuals are those who have been infected with the diseases and then recovered from the infection. With this subdivision, the total population N(t) is governed by

$$N(t)=S(t)+L_{T}(t)+I_{T}(t)+E_{C}(t)+I_{C}(t)+I_{TC}(t)+R(t).$$

In the Omame et al. study [25], they considered vaccinated individuals for COVID-19, and the latent TB-COVID-19 co-infected individuals with in their compartments. They also consider reinfection, but we omit the reinfection of the disease. A second infection is rare as most individuals develop some protection from repeat infections [26]. In our study, we added a population class of COVID-19 latently-infected individuals as they play a major contribution to the prevalence of the virus. In the Omame et al. study, two compartments were given for people recovered from COVID-19, and medicated individuals with TB. Although, we considered a single compartment for the recovered class of people from COVID-19, TB, and their co-infection with different compartmental recovery rates. Some of the brief assumptions to propose TB-COVID-19 coinfection model are reviewed as follows.

We assumed that susceptible individuals are increased by a rate of recruitment Λ and that all population in each compartment are dying with a natural death of *μ*. Besides, susceptible individuals gain TB infections through contact with active TB patients by a force of infection (the incidence is of bilinear mass action) $\lambda_{T}$, given as

(1) $$\lambda_{T}(t)=\beta_{1}\left(I_{T}(t)+I_{TC}(t)\right),$$

where $\beta_{1}$ is the effectual contact rate of the TB bacteria. Moreover, the susceptible individuals also acquire COVID-19 infection, after effective contact with infected individuals of the virus at a force of infection $\lambda_{C}$, expressed as

(2) $$\lambda_{C}(t)=\beta_{2}\left(E_{C}(t)+I_{C}(t)+I_{TC}(t)\right),$$

where $\beta_{2}$ is the effectual contact rate of COVID-19 transmission.

Furthermore, we assumed that individuals pulled out from the TB latent class ($L_{T}$) by growing to a class of active TB with a rate *α* or recover at recovery rate of latent TB infections of *ω*. The latent infected individuals with TB whose immune systems have high may become recovered without the bacteria growing to the active TB infections. While the class of active TB disease ($I_{T}$) become recovered with a rate *γ*, transferred to the co-infection of both TB and COVID-19 at a force of infection $\theta \lambda_{C}$, or die due to TB-induced dying rate of $\delta_{T}$.

Additionally, the TB-COVID-19 co-infected individuals pulled out and move to the $I_{TC}$ compartment at a rate *τ* such that people in the co-infected class may transfer to either the TB only infection by a COVID-19 recovery rate of *nτ* or shift to the COVID-19 infection only at a TB recuperation rate of *mτ*. The population within compartment $I_{TC}$ may die from co-infection induced death rate $\delta_{TC}$ or becomes recovered from both diseases at a rate (1−(m+n)τ).

COVID-19 individuals in exposed class ($E_{C}$) shift the class by becoming infected at a rate *φ* or recover at a rate *π*. In our model, COVID-19 exposed individuals are infectious and may recover without showing any form of the COVID-19 symptoms. Finally, the population in the COVID-19 infected class ($I_{C}$) becomes recovered at a rate *ψ*, transfers to the co-infection class at a force of infection $\nu \lambda_{T}$, or dies from COVID-19 induced death at a rate $\delta_{c}$.

**Figure 1:**
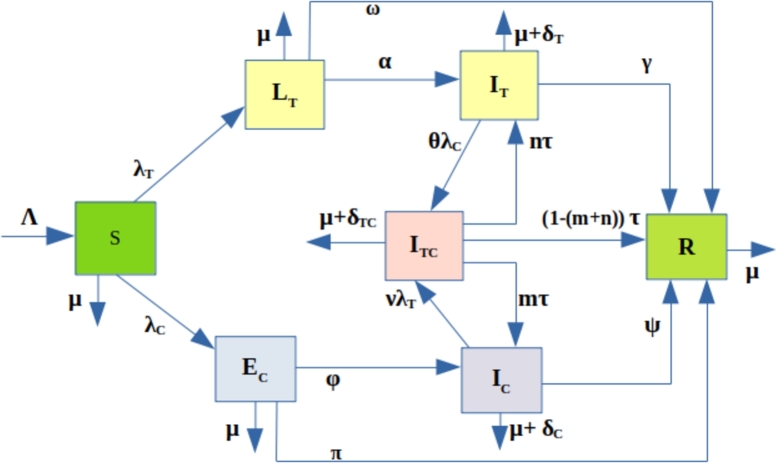
Flow diagram of the model.

The schematic diagram of the proposed model is illustrated in Fig. 1.

Thus, the governing mathematical model can be framed as a system of non-linear differential equations:

(3) $$\left\{ \begin{array}{c} \frac{dS}{dt}=\Lambda -(\lambda_{T}+\lambda_{C}+\mu )S,\\ \frac{dL_{T}}{dt}=\lambda_{T}S-(\mu +\alpha +\omega )L_{T},\\ \frac{dI_{T}}{dt}=\alpha L_{T}+n\tau I_{TC}-(\mu +\delta_{T}+\theta \lambda_{C}+\gamma )I_{T},\\ \frac{dE_{C}}{dt}=\lambda_{C}S-(\mu +\varphi +\pi )E_{C},\\ \frac{dI_{C}}{dt}=\varphi E_{C}+m\tau I_{TC}-(\mu +\delta_{C}+\nu \lambda_{T}+\psi )I_{C},\\ \frac{dI_{TC}}{dt}=\theta \lambda_{C}I_{T}+\nu \lambda_{T}I_{C}-(\mu +\delta_{TC}+\tau )I_{TC},\\ \frac{dR}{dt}=\omega L_{T}+\pi E_{C}+\gamma I_{T}+\psi I_{C}+(1-(m+n))\tau I_{TC}-\mu R, \end{array}\right.$$

where $\lambda_{T}$ and $\lambda_{C}$ are given in the equations (1) and (2), and with initial conditions S(0)>0, $E_{T}(0)\geq 0$, $I_{T}(0)\geq 0$, $E_{C}(0)\geq 0$, $I_{C}(0)\geq 0$, $I_{TC}(0)\geq 0$, and R(0)≥0. The descriptions of model parameters with their values are presented in Table 1.

**Table 1:** Description of the model parameters with their values.

Parameter	Description	Value	Source
Λ	Recruitment rate to the population	2,110,016	calculated
*β*_1_	TB transmission rate	0.6	[21]
*β*_2_	COVID-19 contact rate	0.659	[11]
*α*	Shifting rate of TB exposed individuals to the infected class	0.5	[21]
*ν*	TB coinfection rate from the COVID-19 infections	1.03	assumed
*θ*	COVID-19 coinfection rate from TB infection	1.02	assumed
*π*	Recuperation rate of COVID-19 exposed class	0.716	[11]
*τ*	Rate at which individuals pulled out from *I*_*TH*_	0.025	assumed
*m*	TB recuperation rate of *I*_*TC*_ individuals	0.45	assumed
*n*	COVID-19 recovery rate of *I*_*TC*_ individuals	0.3	assumed
*γ*	Recuperation rate of Active TB infection	0.516	assumed
*δ*_*T*_	Death rate due to TB bacteria	0.001	[21]
*δ*_*C*_	Death rate due to the COVID-19 virus	0.023	[11]
*δ*_*TC*_	Death rate due to TB-COVID-19 coinfection	0.05	assumed
*μ*	Natural dying rate	0.0477	[11]
*φ*	Transfer rate of COVID-19 exposed individuals	0.26	assumed
*ψ*	Recuperation rate of COVID-19 infection	0.475	assumed
*ω*	Recovery rate of latent TB infected class	0.72	[21]

### 2.1 Positivity and boundedness of solutions

Since we are dealing with human populations, all the solutions must be positive and bonded in a feasible region. To assure these, we have the following theorem.

Theorem 2.1 *The solutions of the system of equations*(3)*are positive, unique and bounded in the region*

$$\Omega =\{(S,L_{T},I_{T},E_{C},I_{C},I_{TC},R)\in R_{+}^{7}:0\leq N(t)\leq \frac{\Lambda }{\mu }\}.$$

Proof The functions on the right hand side of the equation (3) are $C^{1}$ on $R_{+}^{7}$. Hence, using the result of Picard–Lindelöf theorem [27], the model equations (3) have a unique solution. For a positively invariant set Ω, and non-negative initial conditions $S(0)>0,E_{T}(0)\geq 0,I_{T}(0)\geq 0,E_{C}(0)\geq 0,I_{C}(0)\geq 0,I_{TC}(0)\geq 0,R(0)\geq 0$, we want to demonstrate that each vector field remains non-negative. Therefore, we have:

(4) $$\frac{dS}{dt}{|}_{S=0}=\Lambda >0,\frac{dL_{T}}{dt}{|}_{L_{T}=0}=\lambda_{T}S\geq 0,\frac{dI_{T}}{dt}{|}_{I_{T}=0}=\alpha L_{T}+n\tau I_{TC}\geq 0,\frac{dE_{C}}{dt}{|}_{E_{C}=0}=\lambda_{C}S\geq 0,\frac{dI_{C}}{dt}{|}_{I_{C}=0}=\varphi E_{C}+m\tau I_{TC}\geq 0,\frac{dI_{TC}}{dt}{|}_{I_{TC}=0}=\theta \lambda_{C}I_{T}+\nu \lambda_{T}I_{C}\geq 0,\frac{dR}{dt}{|}_{R=0}=\omega L_{T}+\pi E_{C}+\gamma I_{T}+\psi I_{C}+(1-(m+n))\tau I_{TC}\geq 0.$$

Hence, following the results of proposition A.1 in [28] and equation (4), the function F(x,t) has the property

$$f_{j}(S,L_{T},I_{T},E_{C},I_{C},L_{TC},I_{TC},R)\geq 0$$

whenever $x\in {\left[0,\infty \right)}^{7},\;x_{j}=0,\;t\geq 0$. As there exists a unique solution for the system of equations (3), it follows that $x(t)\in {\left[0,\infty \right)}^{7}$ for all $t\geq t_{0}\geq 0$ whenever $x(t_{0})\geq 0$.

Besides, the change of total population $N(t)=S+L_{T}+I_{T}+E_{C}+I_{C}+I_{TC}+R$ at time t is governed by

$$\frac{dN}{dt}=\Lambda -\delta_{T}I_{T}-\delta_{c}I_{c}-\delta_{TC}I_{TC}-\mu N\leq \Lambda -\mu N.$$

Consequently, the solution for this linear first order ode becomes $N(t)\leq N(0)e^{-\mu t}+\frac{\Lambda }{\mu }(1-e^{-\mu t})$. Thus, for the given initial data 0≤N(0), we obtain

$$0\leq N(t)\leq \frac{\Lambda }{\mu }.$$

Hence, the solutions of the governing system of nonlinear differential equations in (3) exist, unique and bounded in the feasible region Ω. This concludes the proof. □

## 3 Model analysis

For better understanding the dynamics of co-infection using the proposed model, we first compute the model equilibrium points and then examine the model dynamics around those stationary points. The detailed analysis will be studied by examining the behavior of the sub-models solutions near the equilibrium points for TB, COVID-19, and their coinfection.

### 3.1 COVID-19 only sub-model

Excluding the TB infections from the TB-COVID-19 coinfection model, the sub-model of COVID-19 is obtained as:

(5) $$\left\{ \begin{array}{c} \frac{dS}{dt}=\Lambda -\beta_{2}(E_{C}+I_{C})S-\mu S=f_{1},\\ \frac{dE_{C}}{dt}=\beta_{2}(E_{C}+I_{C})S-(\mu +\varphi +\pi )E_{C}=f_{2},\\ \frac{dI_{C}}{dt}=\varphi E_{C}-(\mu +\delta_{C}+\psi )I_{C}=f_{3},\\ \frac{dR}{dt}=\pi E_{C}+\psi I_{C}-\mu R=f_{4}. \end{array}\right.$$

#### 3.1.1 Local stability analysis of equilibrium points

All the steady state solutions of the COVID-19 sub-model are computed by solving the right hand side system of equations:

$$\Lambda -\beta_{2}(E_{C}+I_{C})S-\mu S=0,\beta_{2}(E_{C}+I_{C})S-(\mu +\varphi +\pi )E_{C}=0,\varphi E_{C}-(\mu +\delta_{C}+\psi )I_{C}=0,\pi E_{C}+\psi I_{C}-\mu R=0.$$

One of the steady state solutions called disease-free equilibrium point ($E_{0}^{c}$) is computed by setting the diseases states $E_{C}=0$ and $I_{C}=0$. As a result, the disease free equilibrium point is calculated as $E_{0}^{c}=(\frac{\Lambda }{\mu },0,0,0)$.

In studying epidemiological models, the basic reproduction number ($R_{0}$) is one of the crucial parameters, which is defined as the number of secondary infections obtained from a single primarily infected individual. We calculated $R_{0}$, denoted as ($R_{0}^{C}$) of the COVID-19 sub-model using the next generation matrix method [29]. Following the result given in [29], and from Equation (5) we have:

$$F_{i}=\left(\begin{array}{c} \beta_{2}(E_{C}+I_{C})S\\ 0 \end{array}\right),V_{i}^{+}=\left(\begin{array}{c} 0\\ \varphi E_{C} \end{array}\right)\;\text{and}\;V_{i}^{-}=\left(\begin{array}{c} (\mu +\varphi +\pi )E_{C}\\ (\mu +\delta_{C}+\psi )I_{C} \end{array}\right)$$

The Jacobian matrices of F(x) and V(x) are, respectively

$$F=DF(E_{0}^{c})=\left[\begin{array}{cc} \beta_{2}\frac{\Lambda }{\mu }\; & \beta_{2}\frac{\Lambda }{\mu }\\ 0\; & 0 \end{array}\right]\;\text{and}\;V=DV(E_{0}^{c})=\left[\begin{array}{cc} \mu +\varphi +\pi \; & 0\\ -\varphi \; & \mu +\delta_{c}+\psi \end{array}\right].$$

It is easy to calculate the inverse of *V* which is given by

$$V^{-1}=\left[\begin{array}{cc} \frac{1}{\mu +\varphi +\pi }\; & 0\\ \frac{\varphi }{\left(\mu +\varphi +\pi )(\mu +\delta_{c}+\psi \right)}\; & \frac{1}{\mu +\delta_{c}+\psi } \end{array}\right].$$

Hence, the next-generation matrix given by $FV^{-1}$ is

$$FV^{-1}=\left[\begin{array}{cc} \frac{\beta_{2}\Lambda }{\mu (\mu +\varphi +\pi )}\left(1+\frac{\varphi }{\mu +\delta_{c}+\psi }\right)\; & \frac{\beta_{2}\Lambda }{\mu (\mu +\delta_{c}+\psi )}\\ 0\; & 0 \end{array}\right].$$

The eigenvalues of $FV^{-1}$ are $\lambda_{1}=\frac{\beta_{2}\Lambda }{\mu (\mu +\varphi +\pi )}\left(1+\frac{\varphi }{\mu +\delta_{c}+\psi }\right)$, and $\lambda_{2}=0$. Hence, we have the following theorem:

Theorem 3.1 *The basic reproduction number of the COVID-19 only sub-model is*

(6) $$R_{0}^{C}=\frac{\beta_{2}\Lambda }{\mu (\mu +\varphi +\pi )}\left(\frac{\mu +\delta_{c}+\psi +\varphi }{\mu +\delta_{c}+\psi }\right).$$

To examine the local stability analysis of the steady state solutions, we used the linearization approach. The Jacobean matrix of the sub-model (5) is

(7) $$J(S,E_{C},I_{C},R)=\left(\begin{array}{cccc} -\beta_{2}(E_{C}+I_{C})-\mu \; & -\beta_{2}S\; & -\beta_{2}S\; & 0\\ \beta_{2}(E_{C}+I_{C})\; & \beta_{2}S-(\mu +\varphi +\pi )\; & \beta_{2}S\; & 0\\ 0\; & \varphi \; & -(\mu +\delta_{C}+\psi )\; & 0\\ 0\; & \pi \; & \psi \; & -\mu \end{array}\right)$$

Then, the Jacobean matrix in equation (7) of the sub-model at $E_{0}^{C}$ is given by:

(8) $$J(E_{0}^{C})=\left(\begin{array}{cccc} -\mu \; & -\beta_{2}\frac{\Lambda }{\mu }\; & -\beta_{2}\frac{\Lambda }{\mu }\; & 0\\ 0\; & \beta_{2}\frac{\Lambda }{\mu }-(\mu +\varphi +\pi )\; & \beta_{2}\frac{\Lambda }{\mu }\; & 0\\ 0\; & \varphi \; & -(\mu +\delta_{C}+\psi )\; & 0\\ 0\; & \pi \; & \psi \; & -\mu \end{array}\right).$$

Two of the eigenvalues for the matrix equation (8) are $\lambda_{1,2}=-\mu$, and the other two are obtained from the reduced matrix

$$J_{2}=\left(\begin{array}{cc} \beta_{2}\frac{\Lambda }{\mu }-(\mu +\varphi +\pi )\; & \beta_{2}\frac{\Lambda }{\mu }\\ \varphi \; & -(\mu +\delta_{C}+\psi ) \end{array}\right).$$

As $\lambda_{1,2}=-\mu <0$, the other two eigenvalues are negative if the trace of the reduced matrix $J_{2}$ is negative and its determinant is positive.

The trace is $trJ_{2}=\beta_{2}\frac{\Lambda }{\mu }-(2\mu +\varphi +\pi +\psi +\delta_{c})$, and is less than zero if $\beta_{2}\frac{\Lambda }{\mu }<(2\mu +\varphi +\pi +\psi +\delta_{c})$. The determinant is $detJ_{2}=-(\beta_{2}\frac{\Lambda }{\mu }-(\mu +\varphi +\pi ))(\mu +\delta_{C}+\psi )-\varphi \beta_{2}\frac{\Lambda }{\mu }=-\beta_{2}\frac{\Lambda }{\mu }(\mu +\delta_{C}+\psi +\varphi )+(\mu +\varphi +\pi )(\mu +\delta_{C}+\psi )$. The sign of the determinant is positive if $\frac{\beta_{2}\Lambda (\mu +\delta_{C}+\psi +\varphi )}{\mu (\mu +\varphi +\pi )(\mu +\delta_{C}+\psi )}<1$, that is $detJ_{2}>0$ if $R_{0}^{C}<1$ and $detJ_{2}<0$ if $R_{0}^{C}>1$.

As a result, the disease free equilibrium point ($E_{0}^{C}$) of the COVID-19 sub-model is stable if $R_{0}^{C}<1$ and unstable if $R_{0}^{C}>1$.

The disease-existing equilibrium point of the COVID-19 only model is obtained by solving equation (9).

(9) $$\Lambda -(\lambda_{c}+\mu )S=0,\lambda_{c}S-(\mu +\varphi +\pi )E_{C}=0,\varphi E_{C}-(\mu +\delta_{C}+\psi )I_{C}=0,\pi E_{C}+\psi I_{C}-\mu R=0.$$

Let $\Sigma_{c}=(S^{⁎},E_{C}^{⁎},I_{C}^{⁎},R)$, be the endemic equilibrium point. We solve the system of equations in terms of the force of infection ($\lambda_{c}^{⁎}$), given by $\lambda_{c}^{⁎}=\beta_{2}(E_{C}^{⁎}+I_{C}^{⁎})$. After solving the equations of (9), we have the following expressions:

(10) $$S^{⁎}=\frac{\Lambda }{\lambda_{c}^{⁎}+\mu },\;E_{C}^{⁎}=\frac{\lambda_{c}^{⁎}\Lambda }{c_{1}(\lambda_{c}^{⁎}+\mu )},\;I_{C}^{⁎}=\frac{\lambda_{c}^{⁎}\Lambda \varphi }{c_{1}c_{2}(\lambda_{c}^{⁎}+\mu )}\;\;\text{and}\;R^{⁎}=\frac{\lambda_{c}^{⁎}\Lambda (c_{2}\pi +\varphi \psi )}{\mu c_{1}c_{2}(\lambda_{c}^{⁎}+\mu )}$$

where $c_{1}=\mu +\varphi +\pi$ and $c_{2}=\mu +\delta_{c}+\psi$. Using equation (10) in the expression of $\lambda_{C}^{⁎}$ gives: $\lambda_{C}^{⁎}=\frac{\beta_{2}\Lambda (c_{2}+\varphi )-c_{1}c_{2}\mu }{c_{1}c_{2}}$. After simplification, we have

$$\lambda_{c}^{⁎}=\mu \left(\frac{\beta_{2}\Lambda (c_{2}+\varphi )}{\mu c1c2}-1\right)=\mu \left(R_{0}^{C}-1\right).$$

This shows that, the COVID-19 force of infection $\lambda_{C}^{⁎}$ is positive at the disease state equilibrium point, only if $R_{0}^{C}>1$. Thus we have just proved the following.

Theorem 3.2 *For*$R_{0}^{C}>1$*, the COVID-19 sub-model*(5)*has a unique endemic (disease existing) equilibrium point.*

#### 3.1.2 The global stability analysis of $\Sigma_{c}$

We have analyzed the global stability of the disease existing equilibrium point $\Sigma_{c}$ using Lyapunov method. To do these, we have defined the following Lyapunov function:

$$L(S,E_{C},I_{C},R)=\frac{1}{2}{\left((S-S^{⁎})+(E_{c}-E_{c}^{⁎})+(I_{c}-I_{c}^{⁎})+(R-R^{⁎})\right)}^{2}.$$

The Lyapunov function *L* is always positive, and it is equal to zero only at the diseases existing equilibrium point $\Sigma_{c}$. After differentiating the function *L* with time, we have

$$\frac{dL}{dt}=\left((S-S^{⁎})+(E_{c}-E_{c}^{⁎})+(I_{c}-I_{c}^{⁎})+(R-R^{⁎})\right)\;\times \left(\frac{dS}{dt}+\frac{dE_{c}}{dt}+\frac{dI_{c}}{dt}+\frac{dR}{dt}\right)=(N-\frac{\Lambda -\delta_{c}I_{c}^{⁎}}{\mu })(\Lambda -\delta_{c}I_{c}-\mu N)\leq (N-\frac{\Lambda }{\mu })(\Lambda -\mu N)\leq -\frac{{\left(\Lambda -\mu N\right)}^{2}}{\mu }\leq 0.$$

For $R_{0}^{c}>1$, the endemic equilibrium point $\Sigma_{c}$ exists, and hence $\frac{dL}{dt}<0$. This implies that the function *L* is a strictly Lyapunov function which indicates the endemic equilibrium point $\Sigma_{c}$ is globally asymptotically stable. The biological meaning of this result indicates that the COVID-19 have continued to exist in the community for a long period.

#### 3.1.3 Sensitivity analysis of COVID-19 sub-model

In this subsection, we examined the analysis of sensitive parameters of the COVID-19 sub-model. For a parameter *p*, the sensitivity of *p* is defined as how the model behavior to a small change in a parameter value [30], and is given by:

$$S_{p}=\frac{\partial R_{0}^{C}}{\partial p}\frac{p}{R_{0}^{C}},\;\text{where}\;R_{0}^{C}=\frac{\beta_{2}\Lambda }{\mu (\mu +\varphi +\pi )}\left(\frac{\mu +\delta_{C}+\psi +\varphi }{\mu +\delta_{C}+\psi }\right).$$

The sensitivity analysis for each of the sub-model equation (5) parameters with respect to $R_{0}^{C}$ is given by:

$$S_{\beta_{2}}=\frac{\partial R_{0}^{C}}{\partial \beta_{2}}\frac{\beta_{2}}{R_{0}^{C}}=1,S_{\Lambda }=\frac{\partial R_{0}^{C}}{\partial \Lambda }\frac{\Lambda }{R_{0}^{C}}=1,S_{\mu }=\frac{\partial R_{0}^{C}}{\partial \mu }\frac{\mu }{R_{0}^{C}}=-\mu \left(\frac{1}{\mu +\pi +\varphi }-\frac{1}{\delta_{C}+\mu +\psi +\varphi }+\frac{1}{\delta_{C}+\mu +\psi }\right),S_{\delta_{C}}=\frac{\partial R_{0}^{C}}{\partial \delta_{C}}\frac{\delta_{C}}{R_{0}^{C}}=-\frac{\delta_{c}\varphi }{\left(\delta_{C}+\mu +\psi )(\delta_{C}+\mu +\psi +\varphi \right)},S_{\psi }=\frac{\partial R_{0}^{c}}{\partial \psi }\frac{\psi }{R_{0}^{C}}=-\frac{\psi \varphi }{\left(\delta_{C}+\mu +\psi )(\delta_{C}+\mu +\psi +\varphi \right)},S_{\varphi }=\frac{\partial R_{0}^{c}}{\partial \varphi }\frac{\varphi }{R_{0}^{C}}=-\frac{\varphi (\delta_{C}+\psi -\pi )}{\left(\mu +\pi +\varphi )(\delta_{C}+\mu +\psi +\varphi \right)},S_{\pi }=\frac{\partial R_{0}^{C}}{\partial \pi }\frac{\pi }{R_{0}^{C}}=-\frac{\pi }{\left(\mu +\pi +\varphi \right)}.$$

**Figure 2:**
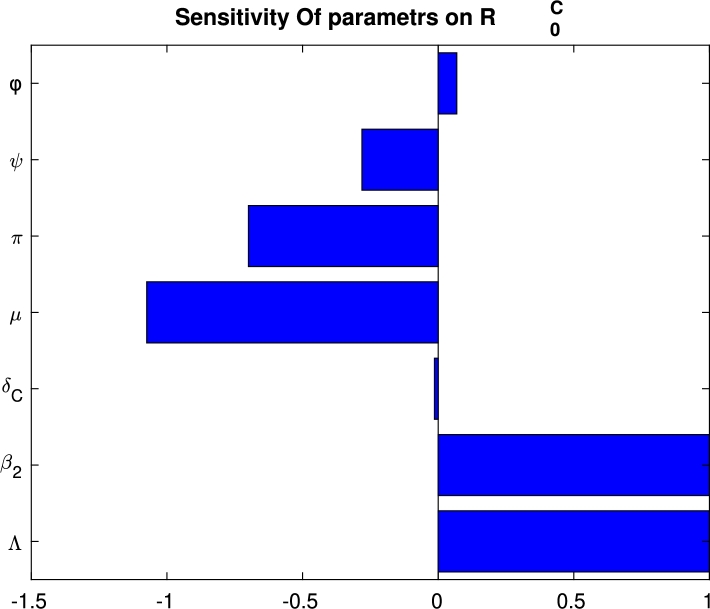
The visual representation of the sensitivity indices of the COVID-19 sub-model basic reproduction number ($R_{0}^{C}$) with respect to parameters.

The numerical values of the sensitivity indices for the COVID-19 sub-model parameters are found in Table 2. The sensitivity analysis of the sub-model revealed that the COVID-19 contact rate $\beta_{2}$, and the recruitment rate Λ have a high positive impact on the spread of the virus. The analysis recommends that the magnitudes of the impacts of Λ and $\beta_{2}$ on the spread of COVID-19 are the same. This is because the number of secondary infections increase with respect to increasing these parameters [30]. The transfer rate of individuals from exposure to an infected class *φ* has a positive impact on the spread of the virus. The other parameters, such as *μ*, *ψ*, $\delta_{C}$ and *π*, have a negative impact, which means that increasing the value of such parameters will decrease the number of people infected with COVID-19. A graphical representation for the sensitivity indices of $R_{0}^{C}$ is illustrated in Fig. 2.

**Table 2:** The sensitivity indices for COVID-19 only sub-model by plugging the parameter values from Table 1.

Parameter	Description	Sensitivity Indices
Λ	Recruitment rate	+1
*β*_2_	COVID-19 contact rate	+1
*μ*	Natural death rate	−0.9748
*δ*_*C*_	COVID-19 induced death rate	−0.0136
*φ*	Transfer rate from exposed to infected class	+0.0687
*π*	Exposed COVID-19 recuperation rate	−0.6994
*ψ*	Recuperation rate of infected COVID-19	−0.2809

### 3.2 TB sub-model

The sub-model of equation (3) with no COVID-19 disease, is given by

(11) $$\frac{dS}{dt}=\Lambda -\beta_{1}I_{T}S-\mu S,\frac{dL_{T}}{dt}=\beta_{1}I_{T}S-(\mu +\alpha +\omega )L_{T},\frac{dI_{T}}{dt}=\alpha L_{T}-(\mu +\delta_{T}+\gamma )I_{T},\frac{dR}{dt}=\omega L_{T}+\gamma I_{T}-\mu R.$$

The disease-free equilibrium point of the TB sub-model equation (11) is given by $E_{0}^{T}=\left(\frac{\Lambda }{\mu },0,0,0\right)$ and the basic reproduction number of the TB only model is obtained as:

(12) $$R_{0}^{T}=\frac{\alpha \beta_{1}\Lambda }{\mu (\mu +\alpha +\omega )(\mu +\delta_{T}+\gamma )}.$$

Theorem 3.3 *The disease-free equilibrium point (*$E_{0}^{T}$*) of the TB sub-model is locally stable if*$R_{0}^{T}<1$*.*

Proof Using the linearizing approach, the Jacobean matrix of the TB only model at $E_{0}^{T}$ is given by

$$J(E_{0}^{T})=\left[\begin{array}{cccc} -\mu \; & 0\; & -\beta_{1}\frac{\Lambda }{\mu }\; & 0\\ 0\; & -(\mu +\alpha +\omega )\; & \beta_{1}\frac{\Lambda }{\mu }\; & 0\\ 0\; & \alpha \; & -(\mu +\delta_{T}+\gamma )\; & 0\\ 0\; & \omega \; & \gamma \; & -\mu \end{array}\right].$$

We can observe that two of the eigenvalues of $J(E_{0}^{T})$ are −*μ*, and the other two are obtained from the reduced matrix

$$J_{3}=\left[\begin{array}{cc} -(\mu +\alpha +\omega )\; & \beta_{1}\frac{\Lambda }{\mu }\\ \alpha \; & -(\mu +\delta_{T}+\gamma ) \end{array}\right].$$

The trace of the reduced matrix $J_{3}$ is $tr(J_{3})=-(2\mu +\alpha +\omega +\delta_{T}+\gamma )<0$, and the determinant of $J_{3}$ is $det(J_{3})=(\mu +\alpha +\omega )(\mu +\delta_{T}+\gamma )-\alpha \beta_{1}\frac{\Lambda }{\mu }$. So the determinant is positive if $R_{0}^{T}<1$. Hence, the stationary point without the disease state of the TB sub-model is locally asymptotically stable if $R_{0}^{T}<1$. □

We have also obtained the endemic (disease existing) equilibrium point of the TB sub-model by solving the following given system of equations:

$$\Lambda -\lambda_{T}S-\mu S=0,\lambda_{T}S-(\mu +\alpha +\omega )L_{T}=0,\alpha L_{T}-(\mu +\delta_{T}+\gamma )I_{T}=0,\omega L_{T}+\gamma I_{T}-\mu R=0,$$

where $\lambda_{T}=\beta_{1}I_{T}$. After solving the given system, we obtain:

$S^{⁎}=\frac{\Lambda }{\lambda_{T}^{⁎}+\mu }$, $L_{T}^{⁎}=\frac{\lambda_{T}^{⁎}\Lambda }{k_{1}(\lambda_{T}^{⁎}+\mu )}$, $I_{T}^{⁎}=\frac{\alpha \lambda_{T}^{⁎}\Lambda }{k_{1}k_{2}(\lambda_{T}^{⁎}+\mu )}$, and $R^{⁎}=\frac{\lambda_{T}^{⁎}\Lambda (\omega k_{2}+\alpha \gamma )}{k_{1}k_{2}\mu (\lambda_{T}^{⁎}+\mu )}$. Using the solutions of $S^{⁎}$, $L_{T}^{⁎}$, $I_{T}^{⁎}$ and $R^{⁎}$, the simplified equation for $\lambda_{T}$ becomes $\lambda_{T}^{⁎}=\frac{\alpha \beta_{1}\Lambda -k_{1}k_{2}\mu }{k_{1}k_{2}}=\mu \left(\frac{\alpha \beta_{1}\Lambda }{k_{1}k_{2}}-1\right)$. That gives $\lambda_{T}^{⁎}=\mu (R_{0}^{T}-1)$, so the endemic equilibrium point exists if and only if $R_{0}^{T}>1$.

Theorem 3.4 *The disease existing equilibrium point of the TB only model is globally stable if* $R_{0}^{T}>1$ *.*

Proof We define the Lyapunov function as follows:

$$V(S,L_{T},I_{T},R)=\frac{1}{2}{\left((S-S^{⁎})+(L_{T}-L_{T}^{⁎})+(I_{T}-I_{T}^{⁎})+(R-R^{⁎})\right)}^{2}.$$

Now we want to show that the proposed function *V* is a Lyapunov function. Differentiating the given function *V* with time, we have

$$\frac{dV}{dt}=\left((S-S^{⁎})+(L_{T}-L_{T}^{⁎})+(I_{T}-I_{T}^{⁎})+(R-R^{⁎})\right)\;\times \left(\frac{dS}{dt}+\frac{dL_{T}}{dt}+\frac{dI_{T}}{dt}+\frac{dR}{dt}\right)=(N-\frac{\Lambda -\delta_{T}I_{T}^{⁎}}{\mu })(\Lambda -\delta_{T}I_{T}-\mu N)\leq (N-\frac{\Lambda }{\mu })(\Lambda -\mu N)\leq -\frac{{\left(\Lambda -\mu N\right)}^{2}}{\mu }\leq 0.$$

Assume that $R_{0}^{T}>1$, for which the endemic equilibrium point exists, then $\frac{dV}{dt}<0$ which imply the function is a strictly Lyapunov function which indicates that the disease existing equilibrium point becomes globally stable. □

#### 3.2.1 Sensitivity analysis of TB sub-model

The sensitivity analysis for the basic reproduction number of the TB sub-model parameters given in equation (5) using normalized forward sensitivity index of its basic reproduction number [30], [31] is given by:

$$S_{\beta_{1}}=\frac{\partial R_{0}^{T}}{\partial \beta_{2}}\frac{\beta_{2}}{R_{0}^{T}}=1,S_{\Lambda }=\frac{\partial R_{0}^{T}}{\partial \Lambda }\frac{\Lambda }{R_{0}^{T}}=1,S_{\mu }=\frac{\partial R_{0}^{T}}{\partial \mu }\frac{\mu }{R_{0}^{T}}=-\frac{\left(2\mu +\alpha +\omega )(\mu +\delta_{T}+\gamma )+\mu (\mu +\alpha +\omega \right)}{\left(\mu +\alpha +\omega )(\mu +\delta_{T}+\gamma \right)},S_{\delta_{T}}=\frac{\partial R_{0}^{T}}{\partial \delta_{T}}\frac{\delta_{T}}{R_{0}^{T}}=-\frac{\delta_{T}}{\delta_{T}+\gamma +\mu },S_{\alpha }=\frac{\partial R_{0}^{T}}{\partial \alpha }\frac{\alpha }{R_{0}^{T}}=\frac{\mu +\omega }{\alpha +\mu +\omega },S_{\omega }=\frac{\partial R_{0}^{T}}{\partial \omega }\frac{\omega }{R_{0}^{T}}=-\frac{\omega }{\alpha +\mu +\omega },S_{\gamma }=\frac{\partial R_{0}^{T}}{\partial \gamma }\frac{\gamma }{R_{0}^{T}}=-\frac{\gamma }{\delta_{T}+\gamma +\mu }.$$

From the sensitivity indices Table 3, we can observe that the TB contact rate $\beta_{1}$, and the recruitment rate Λ have a high positive impact on the spread of the disease. The transfer rate of individuals from latent to infected class *α* has a positive impact for its spread. The other parameters, such as *μ*, *ω*, $\delta_{T}$ and *γ*, have a negative impact, which means increasing the value of such parameters will decrease the number of people infected with TB. For instance, a 1% increase in the TB recovery rate *γ* will produce 0.85% decrease in its basic reproduction number $R_{0}^{T}$.

**Table 3:** The sensitivity indices for TB only sub-model by plugging the parameter values from Table 1.

Parameter	Description	Sensitivity Indices
Λ	Recruitment rate	+1
*β*_1_	TB contact rate	+1
*μ*	Natural death rate	−0.7227
*δ*_*T*_	TB induced death rate	−0.0678
*α*	Transfer rate from latent to active TB	+0.7058
*ω*	Latent TB recovery rate	−0.6619
*γ*	Active TB recovery rate	−0.8533

### 3.3 Analysis of the co-infection model

We then calculated the equilibrium points of the full model (3) by solving the right hand side of the model equations (13):

(13) $$\Lambda -(\lambda_{T}+\lambda_{C}+\mu )S=0,\lambda_{T}S-(\mu +\alpha +\omega )L_{T}=0,\alpha L_{T}+n\tau I_{TC}-(\mu +\theta \lambda_{c}+\delta_{T}+\gamma )I_{T}=0,\lambda_{C}S-(\mu +\varphi +\pi )E_{C}=0,\varphi E_{C}+m\tau I_{TC}-(\mu +\delta_{C}+\nu \lambda_{T}+\psi )I_{C}=0,\theta \lambda_{C}I_{T}+\nu \lambda_{T}I_{C}-(\mu +\delta_{TC}+\tau )I_{TC}=0,\omega L_{T}+\pi E_{C}+\gamma I_{T}+\psi I_{C}+(1-(m+n))\tau I_{TC}-\mu R=0,$$

where the force of infections $\lambda_{T}$ and $\lambda_{C}$ are same with the equations (1) and (2). The disease free equilibrium point ($E_{0}$) of the full model is then obtained as:

$$E_{0}=\left(\frac{\Lambda }{\mu },0,0,0,0,0,0\right).$$

Now, using the next generation matrix [29], we have calculated the basic reproduction number ($R_{0}$) of the full model as follows. Using the notation of the diseased states $X=(L_{T},I_{T},E_{C},I_{C},I_{TC})$, we have the functions in vector form:

$$F(X)=\left[\begin{array}{c} \lambda_{T}S\\ 0\\ \lambda_{c}S\\ 0\\ \theta \lambda_{C}I_{T}+\nu \lambda_{T}I_{C} \end{array}\right]\;\text{and}\;V(X)=\left[\begin{array}{c} (\mu +\alpha +\omega )L_{T}\\ (\mu +\delta_{T}+\gamma )I_{T}-\alpha L_{T}-n\tau I_{TC}\\ (\mu +\varphi +\pi )E_{C}\\ (\mu +\delta_{C}+\psi )I_{C}-\varphi E_{C}-m\tau I_{TC}\\ (\mu +\delta_{TC}+\tau )I_{TC} \end{array}\right],$$

respectively representing the aspect of new infections, and the transfer of infected people into and out of the compartments. Hence, the Jacobian matrices of F(X) and V(X) at the stationary point $E_{0}$ respectively are,

$$F=DF(E_{0})=\left[\begin{array}{ccccc} 0\; & \beta_{1}\frac{\Lambda }{\mu }\; & 0\; & 0\; & \beta_{1}\frac{\Lambda }{\mu }\\ 0\; & 0\; & 0\; & 0\; & 0\\ 0\; & 0\; & \beta_{2}\frac{\Lambda }{\mu }\; & \beta_{2}\frac{\Lambda }{\mu }\; & \beta_{2}\frac{\Lambda }{\mu }\\ 0\; & 0\; & 0\; & 0\; & 0\\ 0\; & 0\; & 0\; & 0\; & 0 \end{array}\right],\;\text{and},$$

$$V=DV(E_{0})=\left[\begin{array}{ccccc} c_{1}\; & 0\; & 0\; & 0\; & 0\\ -\alpha \; & c_{2}\; & 0\; & 0\; & -n\tau \\ 0\; & 0\; & c_{3}\; & 0\; & 0\\ 0\; & 0\; & -\varphi \; & c_{4}\; & -m\tau \\ 0\; & 0\; & 0\; & 0\; & c_{5} \end{array}\right],$$

where $c_{1}=\alpha +\mu +\omega$, $c_{2}=\delta_{T}+\mu +\gamma$, $c_{3}=\varphi +\mu +\pi$, $c_{4}=\delta_{C}+\mu +\psi$, $c_{5}=\delta_{TC}+\mu +\tau$. Then, the next-generation matrix $FV^{-1}$ is:

$$FV^{-1}=\left[\begin{array}{ccccc} \frac{\alpha \beta_{1}\Lambda }{\mu c_{1}c_{2}}\; & \frac{\beta_{1}\Lambda }{\mu c_{2}}\; & 0\; & 0\; & \frac{\Lambda \beta_{1}(c_{2}+n\tau )}{\mu c_{2}c_{5}}\\ 0\; & 0\; & 0\; & 0\; & 0\\ 0\; & 0\; & \frac{\beta_{2}\Lambda }{\mu c_{3}}\left(\frac{c_{4}+\varphi }{c_{4}}\right)\; & \frac{\beta_{2}\Lambda }{\mu c_{4}}\; & \frac{\Lambda \beta_{2}(c_{4}+m\tau )}{\mu c_{4}c_{5}}\\ 0\; & 0\; & 0\; & 0\; & 0\\ 0\; & 0\; & 0\; & 0\; & 0 \end{array}\right].$$

The three of the eigenvalues for the matrix $FV^{-1}$ are zeros. The other two eigenvalues are:

$$\lambda_{1}=\frac{\alpha \beta_{1}\Lambda }{\mu c_{1}c_{2}}=R_{0}^{T},\;\text{and}\;\lambda_{2}=\frac{\beta_{2}\Lambda }{\mu c_{3}}\left(\frac{c_{4}+\varphi }{c_{4}}\right)=R_{0}^{C}.$$

Hence, the basic reproduction number ($R_{0}$) of the full model (3) is given by

$$R_{0}=\max\{R_{0}^{C},R_{0}^{T}\}.$$

#### 3.3.1 Local stability of $E_{0}$ for the full model

We performed the local stability analysis of the disease free equilibrium point of the full model using the method of linearization. The Jacobean matrix of the full model equation (3) at $E_{0}$ is given in matrix equation (14) by:

(14) $$J(E_{0})=\left(\begin{array}{ccccccc} -\mu \; & 0\; & -\beta_{1}\frac{\Lambda }{\mu }\; & -\beta_{2}\frac{\Lambda }{\mu }\; & -\beta_{2}\frac{\Lambda }{\mu }\; & -(\beta_{1}+\beta_{2})\frac{\Lambda }{\mu }\; & 0\\ 0\; & -c_{1}\; & \beta_{1}\frac{\Lambda }{\mu }\; & 0\; & 0\; & \beta_{1}\frac{\Lambda }{\mu }\; & 0\\ 0\; & \alpha \; & -c_{2}\; & 0\; & 0\; & n\tau \; & 0\\ 0\; & 0\; & 0\; & \beta_{2}\frac{\Lambda }{\mu }-c_{3}\; & \beta_{2}\frac{\Lambda }{\mu }\; & \beta_{2}\frac{\Lambda }{\mu }\; & 0\\ 0\; & 0\; & 0\; & \varphi \; & -c_{4}\; & m\tau \; & 0\\ 0\; & 0\; & 0\; & 0\; & 0\; & -c_{5}\; & 0\\ 0\; & \omega \; & \gamma \; & \pi \; & \psi \; & (1-(m+n))\tau \; & -\mu \end{array}\right)$$

After expanding the characteristic equation $|\lambda I_{7}-J(E_{0})|=0$ with its first and seventh columns, and the sixth row, we obtain three eigenvalues $\lambda_{1,2}=-\mu$, and $\lambda_{3}=-c_{5}$. We will then calculate the remaining four eigenvalues from the reduced matrix

$$(J_{4}-\lambda I_{4})=\left(\begin{array}{cccc} -c_{1}-\lambda \; & \beta_{1}\frac{\Lambda }{\mu }\; & 0\; & 0\\ \alpha \; & -c_{2}-\lambda \; & 0\; & 0\\ 0\; & 0\; & \beta_{2}\frac{\Lambda }{\mu }-c_{3}-\lambda \; & \beta_{2}\frac{\Lambda }{\mu }\\ 0\; & 0\; & \varphi \; & -c_{4}-\lambda \end{array}\right).$$

Hence the remaining eigenvalues are the roots of the reduced characteristic polynomial:

$$X_{4}(\lambda )=\lambda^{4}+\left(c_{1}+c_{2}+c_{3}+c_{4}-\frac{\beta_{2}\Lambda }{\mu }\right)\lambda^{3}\;\;+(c_{1}(c_{2}+c_{3}+c_{4})+c_{2}(c_{3}+c_{4})+c_{3}c_{4}-\frac{\beta_{2}\Lambda }{\mu }(c_{1}+c_{2}+c_{4})\;-\frac{\beta_{1}\Lambda \alpha }{\mu }-\frac{\beta_{2}\Lambda \varphi }{\mu })\lambda^{2}\;\;+(c_{1}c_{2}(c_{3}+c_{4})+c_{3}c_{4}(c_{1}+c_{2})\;-\frac{\beta_{2}\Lambda }{\mu }(c_{4}(c_{1}+c_{2})+\varphi (c_{1}+c_{2})+c_{1}c_{2}+\frac{\beta_{1}\Lambda \alpha }{\mu })\;-\frac{\beta_{1}\Lambda \alpha }{\mu }(c_{3}+c_{4}))\lambda \;\;+c_{4}(c_{3}-\frac{\beta_{2}\Lambda }{\mu })(c_{1}c_{2}-\frac{\beta_{1}\Lambda \alpha }{\mu })-\frac{\beta_{2}\Lambda \varphi }{\mu }(c_{1}c_{2}-\frac{\beta_{1}\Lambda \alpha }{\mu }).$$

That is, four of the eigenvalues are roots of the equation:

(15) $$X_{4}(\lambda )=\lambda^{4}+D_{1}\lambda^{3}+D_{2}\lambda^{2}+D_{3}\lambda +D_{4}=0,$$

where,

$$D_{1}=c_{1}+c_{2}+c_{3}+c_{4}-\frac{\beta_{2}\Lambda }{\mu },=c_{1}+c_{2}+c_{3}+c_{3}c_{4}\left(1-\frac{\beta_{2}\Lambda }{\mu c_{3}c_{4}}\right)=c_{1}+c_{2}+c_{3}+c_{3}c_{4}(1-R_{0}^{C}),D_{2}=c_{1}(c_{2}+c_{3}+c_{4})+c_{2}(c_{3}+c_{4})+c_{3}c_{4}\;\;-\frac{\beta_{2}\Lambda }{\mu }(c_{1}+c_{2}+c_{4})-\frac{\beta_{1}\Lambda \alpha }{\mu }-\frac{\beta_{2}\Lambda \varphi }{\mu },D_{3}=c_{1}c_{2}(c_{3}+c_{4})+c_{3}c_{4}(c_{1}+c_{2})\;-\frac{\beta_{2}\Lambda }{\mu }\left(c_{4}(c_{1}+c_{2})+\varphi (c_{1}+c_{2})+c_{1}c_{2}+\frac{\beta_{1}\Lambda \alpha }{\mu }\right)\;-\frac{\beta_{1}\Lambda \alpha }{\mu }(c_{3}+c_{4}),\;\text{and}\;D_{4}=c_{4}\left(c_{3}-\frac{\beta_{2}\Lambda }{\mu }\right)\left(c_{1}c_{2}-\frac{\beta_{1}\Lambda \alpha }{\mu }\right)-\frac{\beta_{2}\Lambda \varphi }{\mu }\left(c_{1}c_{2}-\frac{\beta_{1}\Lambda \alpha }{\mu }\right),=\left(c_{1}c_{2}-\frac{\beta_{1}\Lambda \alpha }{\mu }\right)\left(c_{3}c_{4}-\frac{\beta_{2}\Lambda }{\mu }(c_{4}+\varphi )\right)=c_{1}c_{2}\left(1-\frac{\beta_{1}\Lambda \alpha }{\mu c_{1}c_{2}}\right)c_{3}c_{4}\left(1-\frac{\beta_{2}\Lambda }{\mu c_{3}c_{4}}(c_{4}+\varphi )\right)=c_{1}c_{2}c_{3}c_{4}\left(1-R_{0}^{T}\right)\left(1-R_{0}^{C}(c_{4}+\varphi )\right).$$

Hence, using Routh-Hurwitz stability conditions, the roots of the characteristic equation (15) of the reduced matrix have negative real parts provided that the following holds.

(16) $$D_{1}>0,\;D_{3}>0,\;D_{4}>0,\;\text{and}\;\;D_{1}D_{2}D_{3}>D_{3}^{2}+D_{1}^{2}D_{4}.$$

It can be noted that the conditions in equation (16) hold if $R_{0}<1$. Thus we have already proved the following.

Theorem 3.5 *The disease free equilibrium point (*$E_{0}$*) of the full model*(3)*becomes locally asymptotically stable provided that*$R_{0}<1$*, and the condition given in equation*(16)*holds.*

#### 3.3.2 Global stability of $\left(E_{0}\right)$ of the full model

Rewriting our model equation (3) as

$$\left\{ \begin{array}{c} \frac{dX}{dt}=F(X,Z)\\ \frac{dZ}{dt}=G(X,Z),\;G(X,0)=0, \end{array}\right.$$

where X=(S,R) and $Z=(L_{T},I_{T},E_{C},I_{C},I_{TC})$, with $X\in R_{+}^{2}$ denoting the healthy individual compartments and $Z\in R_{+}^{5}$ denoting the infected population [32]. Hereafter the disease-free equilibrium state is denoted as

$$U_{0}=(X_{0},0),\;\text{where},\;X_{0}=(\frac{\Lambda }{\mu },0).$$

The conditions $\left(H_{1}\right)$ and $\left(H_{2}\right)$ below guarantee the global asymptotically stability of $E_{0}$ for $R_{0}<1$:

$\left(H_{1}\right)$ For $\frac{dX}{dt}=F(X,0)$, the equilibrium point $U_{0}$ is globally stable;
$\left(H_{2}\right)$ $G(X,Z)=AZ-\overset{ˆ}{G}(X,Z),\;\overset{ˆ}{G}(X,Z)\geq 0$ for (X,Z)∈Ω, where $A=D_{Z}G(U_{0},0)$ is a Metzler matrix, and Ω is the feasible region of the developed model.

From our equation of the co-infection mathematical model (3), we have:

$$\frac{dX}{dt}=F(X,Z)=\left[\begin{array}{c} \Lambda -(\lambda_{T}+\lambda_{C}+\mu )S\\ \omega L_{T}+\pi E_{C}+\gamma I_{T}+\psi I_{C}+(1-(m+n))\tau I_{TC}-\mu R \end{array}\right],$$

$$\text{Hence}\;F(X,0)=\left[\begin{array}{c} \Lambda -\mu S\\ 0 \end{array}\right],\;\text{and}$$

$$\frac{dZ}{dt}=G(X,Z)=\left[\begin{array}{c} \lambda_{T}S-(\mu +\alpha +\omega )L_{T}\\ \alpha L_{T}+n\tau I_{TC}-(\mu +\theta \lambda_{c}+\delta_{T}+\gamma )I_{T}\\ \lambda_{C}S-(\mu +\varphi +\pi )E_{C}\\ \varphi E_{C}+m\tau I_{TC}-(\mu +\delta_{C}+\nu \lambda_{T}+\psi )I_{C}\\ \theta \lambda_{C}I_{T}+\nu \lambda_{T}I_{C}-(\mu +\delta_{TC}+\tau )I_{TC} \end{array}\right].$$

Therefore,

$$A=D_{Z}G(U_{0},0)=\left[\begin{array}{ccccc} -c_{1}\; & \beta_{1}\; & 0\; & 0\; & \beta_{1}\\ \alpha \; & -c_{2}\; & 0\; & 0\; & n\tau \\ 0\; & 0\; & \beta_{2}-c_{3}\; & \beta_{2}\; & \beta_{2}\\ 0\; & 0\; & \varphi \; & -c_{4}\; & m\tau \\ 0\; & 0\; & 0\; & 0\; & -c_{5} \end{array}\right]\;\text{which is a Metzler Matrix}.$$

Here $\overset{ˆ}{G}(X,Z)=AZ-G(X,Z)$, and so,

$$\overset{ˆ}{G}(X,Z)=\left[\begin{array}{c} \overset{ˆ}{G_{1}}(X,Z)\\ \overset{ˆ}{G_{2}}(X,Z)\\ \overset{ˆ}{G_{3}}(X,Z)\\ \overset{ˆ}{G_{4}}(X,Z)\\ \overset{ˆ}{G_{5}}(X,Z) \end{array}\right]=\left[\begin{array}{c} \lambda_{T}(1-S)\\ \theta \lambda_{C}I_{T}\\ \lambda_{C}(1-S)\\ \nu \lambda_{T}I_{C}\\ -\theta \lambda_{C}I_{T}-\nu \lambda_{T}I_{C} \end{array}\right].$$

Hence $\overset{ˆ}{G_{5}}(X,Z)=-\theta \lambda_{C}I_{T}-\nu \lambda_{T}I_{C}<0$, the necessary condition ($H_{2}$) is not satisfied. As a result, $U_{0}$ and then the disease free equilibrium point $E_{0}$ may not be globally asymptotic stable. This indicates a backward bifurcation will occur at $R_{0}=1$. The biological meaning of a backward bifurcation is that a stable disease-state equilibrium point will co-exist with a stable disease-free stationary point provided that the basic reproduction number is less than unity.

### 3.4 Impacts of TB on COVID-19

By first expressing the basic reproduction number of COVID-19 sub-model $R_{0}^{C}$ in terms of $R_{0}^{T}$, we have analyzed the impact of TB disease on the speeding up of COVID-19 pandemic. For this, we began by expressing the parameter Λ (as it is common for both of the equilibrium points) in the equation (12) in terms of $R_{0}^{T}$, we have

$$R_{0}^{T}=\frac{\alpha \beta_{1}\Lambda }{\mu (\mu +k_{1})(\mu +k_{2})},\;\text{where}\;\;k_{1}=\alpha +\omega ,\;\;k_{2}=\delta_{T}+\gamma .$$

Solving for Λ gives

$$\Lambda =\frac{R_{0}^{T}\mu (\mu +k_{1})(\mu +k_{2})}{\alpha \beta_{1}}.$$

Substituting the expression of Λ in $R_{0}^{C}$ (from the equation (6)), we have obtained $R_{0}^{C}$ in terms of $R_{0}^{T}$ as

$$R_{0}^{C}=\frac{\beta_{2}R_{0}^{T}}{\mu (\mu +k_{4})}\left(\frac{\mu +k_{3}+\varphi }{\mu +k_{3}}\right)\left(\frac{\mu (\mu +k_{1})(\mu +k_{2})}{\alpha \beta_{1}}\right)$$

where, $k_{3}=\delta_{C}+\psi$, and $k_{4}=\varphi +\pi$.

The partial derivative of $R_{0}^{C}$ with respect to $R_{0}^{T}$ is then given by

$$\frac{\partial R_{0}^{C}}{\partial R_{0}^{T}}=\frac{\beta_{2}}{\mu (\mu +k_{4})}\left(\frac{\mu +k_{3}+\varphi }{\mu +k_{3}}\right)\left(\frac{\mu (\mu +k_{1})(\mu +k_{2})}{\alpha \beta_{1}}\right)$$

Here, we have observed that the partial derivative of $R_{0}^{C}$ with respect to $R_{0}^{T}$ is positive. This result indicates that an increase of the TB infection within the population will positively influence for the spread of the COVID-19 pandemic.

## 4 Numerical simulation

In the above sections, we have discussed the analytical behaviors of the proposed mathematical model (3) and the sub-models. Here, we carry out numerical simulation of the proposed co-infection model using the ode45 package of Matlab software. In doing so, we use the initial values of the state variables for the model equations given in Table 4 and all parameter values illustrated in Table 1.

**Table 4:** The initial values of the state variables in the model equation (3).

State variable	*S*	*L*_*T*_	*I*_*T*_	*E*_*C*_	*I*_*C*_	*I*_*TC*_	*R*
Initial Values	66454111	33220000	152005	495424	123856	700	220000

We take these initial values for the COVID-19 infected and recovered compartments reasonably to the same as the observed data of WHO situation reports for the case of Ethiopia at the end of the year 2020. The total Ethiopian population on December 31, 2020, was about 103,724,912 with 123,856 COVID-19 cases [33]. We make the assumption that about 80% of the disease is asymptomatic, which helps us outline the initial value for Exposed COVID-19 class [34], [35]. We also assume that a large percentage of about 33.3% of the population is infected with TB in the latent class. This is from the WHO report that about one-third of the world's population has latent TB [36]. The initial data for the TB compartments were collected from the Ethiopian ministry of Health (MOE) [33].

**Figure 3:**
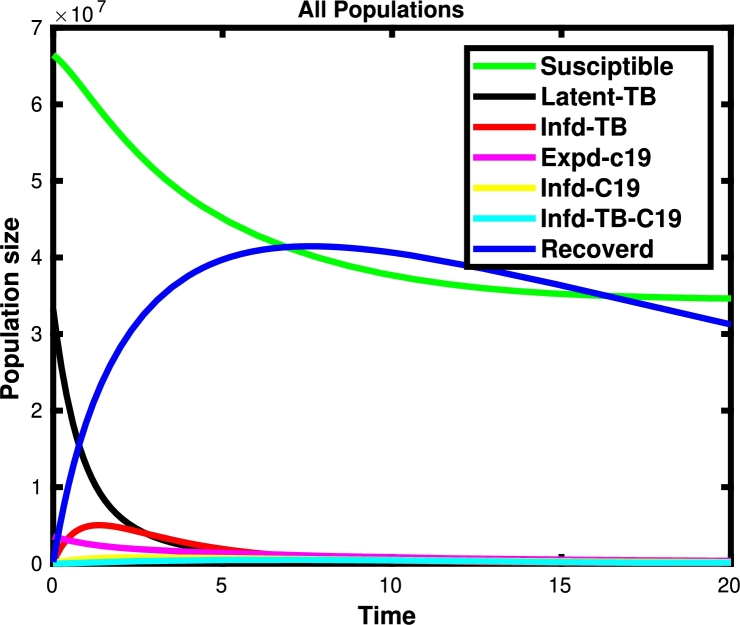
The solutions of full model equation (3), with parameter values given in Table 1.

**Figure 4:**
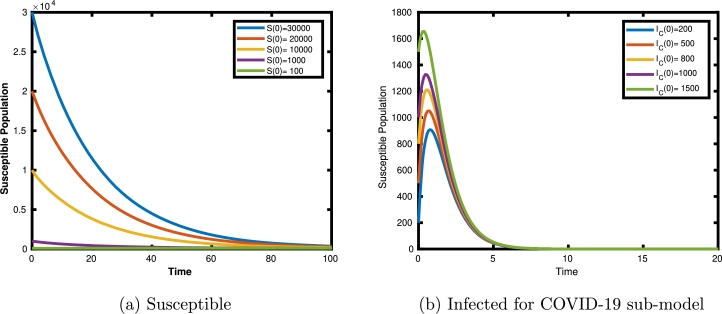
Stability illustrations of the disease free equilibrium point for *R*_0_ < 1, and *R*_0_ > 1.

**Figure 5:**
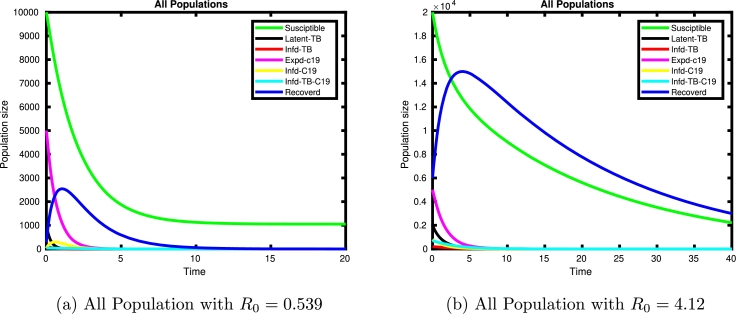
Stability illustrations of the disease free equilibrium point for *R*_0_ < 1, and *R*_0_ > 1.

The time series plots for the numerical solutions of the co-infection model equation (3) are plotted in Fig. 3 with parameter values given in Table 1. Figs. 4a and 4b indicate the stability of the solutions with different initial conditions for the susceptible and infected compartments of the COVID-19 sub-model. Figs. 5a and 5b indicate the stable and unstable part of disease free equilibrium point, which supports our analytical findings.

**Figure 6:**
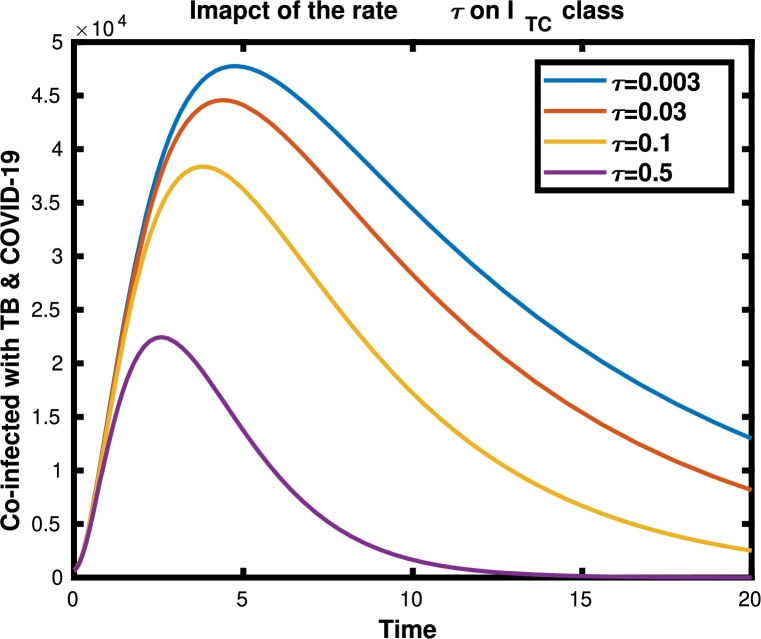
The dynamics of *I*_*TC*_ with the values of *τ* given in the legend, and other parameter values are given in Table 1.

The impact of the rate *τ* (rate at which individuals leave the co-infected class $I_{TC}$) for the co-infected individuals is illustrated in Fig. 6. It was shown that an increase in the rate will decrease the number of individuals in that class.

**Figure 7:**
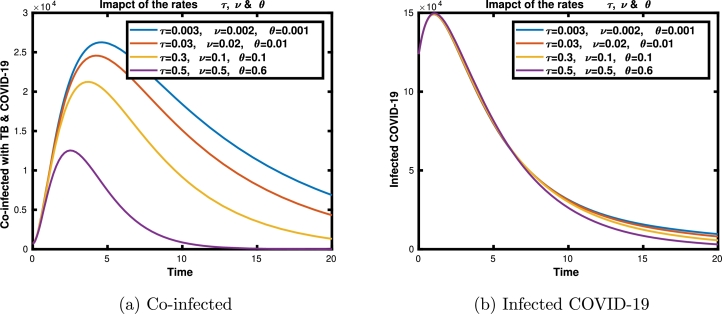
Impacts of *τ*, *ν* and *θ* on the co infected classes (*I*_*TC*_) and on the COVID-19 infected class (*I*_*C*_).

**Figure 8:**
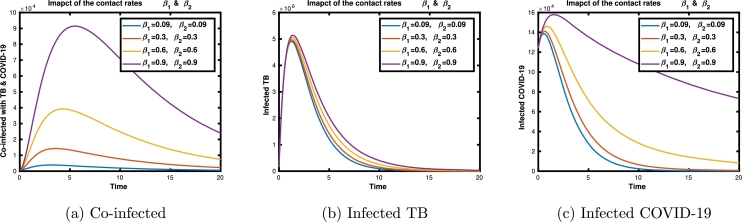
Impacts of the contact rates *β*_1_ and *β*_2_ on the transmission dynamics of infected TB (*I*_*T*_), infected COVID-19 (*I*_*C*_) and the co-infected ones (*I*_*TC*_).

The Figures illustrated in Fig. 7a and 7b show the impact of transfer rates to the co-infected class from each actively infected individual of the diseases. In Fig. 8, we have shown the impacts of the contact rates of disease for infected compartments of our system (3). The spreads of co-infected individuals with varying effective contact rates are shown in Fig. 8a, while the spreads of infected individuals with TB and COVID-19 with different contact rates are observed in Figs. 8b and 8c, respectively. We conclude that the states $I_{T}$, $I_{C}$, and $I_{TC}$ increase as the transmission coefficients increase. All these numerical results support our analytical findings of the sensitivity analysis in sub-models.

## 5 Conclusion

In conclusion, a mathematical model to examine the possible spread of the coinfection of TB and COVID-19 diseases has been proposed. We proved the model solutions positivity and boundedness properties in a biologically feasible region. Additionally, we had computed the equilibrium points of the sub-models, and their stabilities are analyzed with respect to the basic reproduction numbers. In both sub-models, we proved that the disease-free stationary states are stable provided that the respective reproduction numbers are less than one. Whenever the reproduction numbers are greater than unity, the disease-free equilibrium point is unstable, and there exists a unique endemic equilibrium point that is both locally and globally asymptotically stable. Biologically, this result implies that the both diseases will persist in the population for a longer time. We have also computed its reproduction number of the co-infection biological system and analyzed the local and global stability of the disease-free stationary point. Moreover, the results from sensitivity analysis show that an increase in rate of infections from the TB and COVID-19 infected individuals would increase the TB and COVID-19 co-infection. Finally, the results from the numerical simulations imply an effectual decrease of thee contact rates and a simultaneous increase of treatments lead for minimizing the spread of the co-infection of TB and COVID-19, as we had seen in the numerical Figure 6, Figure 7, Figure 8. In extension of the model for future study, we are interesting to observe the individual knowledge about the disease and their behavior changes to protect themselves based on the knowledge they have acquired. Moreover, we want to extend the model to a fractional order derivative form and apply optimal control problems to advise optimal strategies for mitigating the diseases in the community.

## Declarations

### Author contribution statement

Kassahun Getnet Mekonen, Legesse Lemecha Obsu: Conceived and designed the experiments; Performed the experiments; Analyzed and interpreted the data; Contributed reagents, materials, analysis tools or data; Wrote the paper.

### Funding statement

This research did not receive any specific grant from funding agencies in the public, commercial, or not-for-profit sectors.

### Data availability statement

Data included in article/supp. material/referenced in article.

### Declaration of interests statement

The authors declare no conflict of interest.

### Additional information

No additional information is available for this paper.
